# A Method for Human Pose Estimation and Joint Angle Computation Through Deep Learning

**DOI:** 10.3390/jimaging12040157

**Published:** 2026-04-06

**Authors:** Ludovica Ciardiello, Patrizia Agnello, Marta Petyx, Fabio Martinelli, Mario Cesarelli, Antonella Santone, Francesco Mercaldo

**Affiliations:** 1 Department of Medicine and Health Sciences “Vincenzo Tiberio”, University of Molise, 86100 Campobasso, Italy; antonella.santone@unimol.it; 2 Istituto Nazionale per l’Assicurazione Contro gli Infortuni sul Lavoro, 00144 Roma, Italy; p.agnello@inail.it (P.A.); m.petyx@inail.it (M.P.); 3 Institute for High Performance Computing and Networking, National Research Council of Italy (CNR), 87036 Rende, Italy; fabio.martinelli@icar.cnr.it; 4 Department of Engineering, University of Sannio, 82100 Benevento, Italy; mcesarelli@unisannio.it

**Keywords:** human pose estimation, HPE, angle computation, object detection, deep learning, artificial intelligence

## Abstract

Human pose estimation is a crucial task in computer vision with widespread applications in healthcare, rehabilitation, sports, and remote monitoring. In this paper, we propose a deep learning-based method for automatic human pose estimation and joint angle computation, tailored specifically for physiotherapy and telemedicine scenarios. Beyond pose estimation, the proposed method is able to compute angles between joints, enabling analysis of body alignment and posture. The proposed approach is built upon a customized skeleton with 25 anatomical keypoints and a dataset composed of over 150,000 annotated and augmented images derived from multiple open-source datasets. Experimental results demonstrate the effectiveness of the proposed method, achieving a mAP@50 of 0.58 for keypoint localization and 0.98 for object detection. Moreover, we demonstrate several real-world practical use cases in evaluating exercise correctness and identifying postural deviations by exploiting the proposed method, confirming that the proposed method can represent a promising approach for automated motion analysis, with potential impact on digital health, rehabilitation support, and remote patient care.

## 1. Introduction

Human pose estimation (HPE) represents a critical task in computer vision, as a matter of fact it enables machines to interpret human body postures and movements [1]. Automatic HPE models find applications in a plethora of domains, including healthcare, sports analytics, robotics, surveillance, and human-computer interaction [2]. In healthcare, for instance, accurate HPE aids in physical therapy by monitoring patient progress, while in sports, it provides actionable insights for performance optimization and injury prevention [3].

The importance of robust HPE models is particularly evident in the fields of physiotherapy and rehabilitation, where accurate monitoring of body movements is essential for recovery [4]. In physiotherapy, patients undergoing treatment for musculoskeletal injuries rely on precise feedback about their posture and movements [5]. In fact, automatic HPE models can facilitate this by providing real-time assessments of motion and alignment during exercises, ensuring that patients perform movements correctly. This minimizes the risk of exacerbating injuries and accelerates the recovery process.

Similarly, in rehabilitation settings, HPE can offer significant advantages in the assessment and guidance of patients recovering from neurological conditions such as strokes or spinal cord injuries, by monitoring subtle changes in motor function and providing detailed insights into a patient’s progress [6]. By enabling therapists to track rehabilitation outcomes remotely, pose estimation supports more personalized and data-driven recovery plans.

In the context of telemedicine, the role of HPE is increasingly prominent. With the growing adoption of remote healthcare services, pose estimation algorithms enable clinicians to assess patient movements via video consultations [7]. This is particularly valuable in situations where in-person visits are impractical, such as during pandemics or in rural areas with limited access to healthcare facilities. These systems provide patients with the opportunity to engage in supervised exercise sessions from the comfort of their homes, ensuring continuity of care while reducing logistical barriers.

The consequences of incorrect body posture or improper movement patterns highlight the urgency of reliable HPE [8]. Poor posture and incorrect movements are significant contributors to musculoskeletal disorders, repetitive strain injuries, and chronic pain. For instance, an improperly executed therapeutic exercise can aggravate an existing injury or delay recovery [9]. Automated systems that provide accurate feedback on body posture and movement in real-time can mitigate these risks, enhancing the quality and safety of care.

Deep learning has revolutionized the field of HPE [10], offering advanced capabilities for analyzing complex human movements. Notably, Convolutional Neural Networks (CNNs), graph-based models, and transformer architectures demonstrated really interesting performances in recognizing joint positions and spatial relationships [11]. However, achieving robust pose estimation in real-world settings remains a challenge due to factors such as occlusion, diverse body shapes, environmental variability, and the need for low-latency performance [12].

Starting from these considerations, in this paper we propose a deep learning-based method for HPE, with the aim to address these challenges. The method is designed to provide accurate and reliable pose estimation across diverse conditions, making it particularly well-suited for applications in physiotherapy, rehabilitation, and telemedicine.

Furthermore, the proposed method, based on the results of the HPE model, is also aimed to compute the angle formed between the various joints of the body, but also for example between the back and the legs or hands, in order to provide support to the rehabilitation staff by indicating angles that are symptomatic of incorrect postures and which can therefore lead to damage, for example, to the spinal column.

Thus, by improving HPE accuracy, the proposed approach supports the development of models that not only monitor and correct posture but also prevent injury and enhance patient outcomes in both clinical and also remote settings.

Below, we itemize the main contributions of the paper:

- The design of a 25-keypoint skeleton model, providing enhanced detail for hands and feet compared to standard representations.
- The development of an automatic script for computing joint angles between keypoints, enabling straightforward measurement of posture and movement.

The paper proceeds as follows: in Section 2, we present the method we designed and developed for HPE and joint angle computation; the results of the experimental analysis are provided and discussed in Section 3; an overview of the state-of-the-art literature in the HPE context is provided in Section 4; and, finally, conclusion and future research plans are drawn in the Section 6.

## 2. The Method

In this section, we present the method we designed and developed for automatic HPE and joint angle detection.

**Figure 1:**
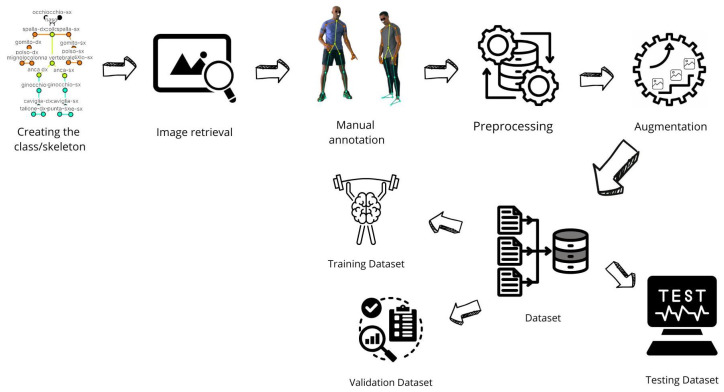
The workflow of the dataset construction phase.

**Figure 2:**
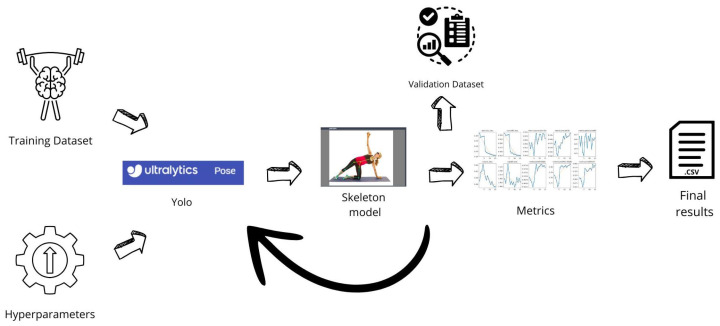
The workflow of the model training phase.

**Figure 3:**
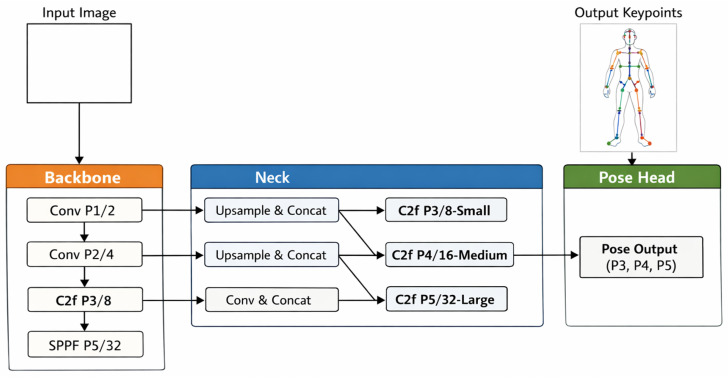
The architecture of the adopted YOLO8n model for HPE.

**Figure 4:**
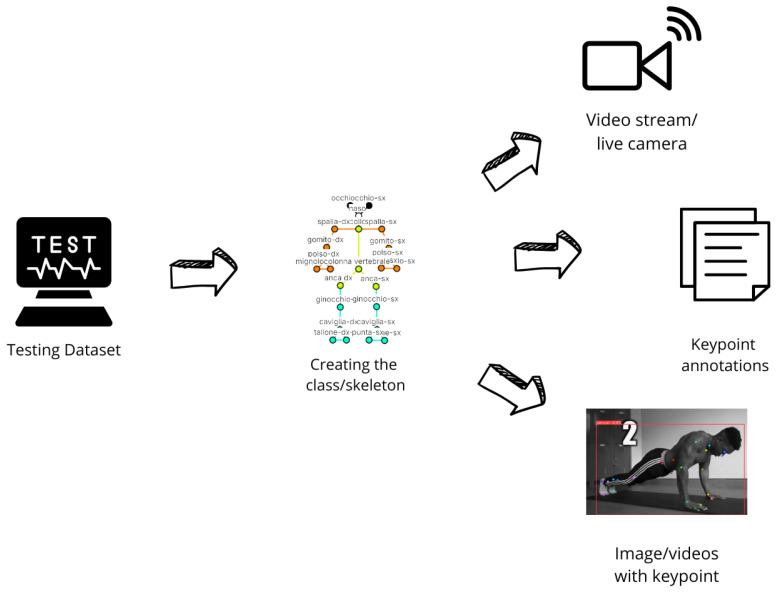
The workflow of the model testing phase.

**Figure 5:**
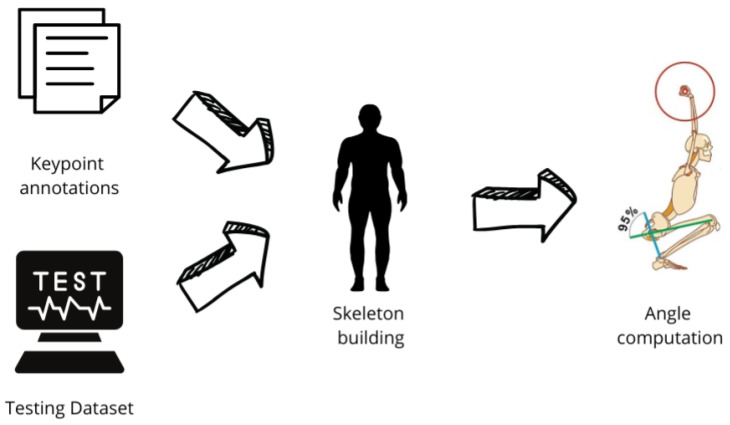
The workflow of the HPE and angle computation step.

The proposed method mainly consists of following four different phases: the first one is related to the Dataset construction (the workflow of which is shown in Figure 1), related to generation of the human skeleton and to dataset building; the second one is related to the Model Training (the workflow of which is shown in Figure 2, while the architecture is depicted in Figure 3) responsible for the training process of the HPE model; the third phase is the Model Testing, shown in Figure 4, related to the testing process of the HPE model built in the previous phase, aimed to confirm the effectiveness of the HPE model; and the last phase, the workflow of which is shown in Figure 5, is represented by the Angle Computation, aimed to compute the angle from the coordinates obtained from the keypoint predictions (obtained from the HPE model). In the following, we describe each phase of the proposed method in a dedicated subsection.

### 2.1. Dataset Construction

To develop an HPE model, it is crucial to create a skeleton/class and an image dataset: this represents the first phase of the proposed method, the workflow of which is shown in Figure 1.

The first step involves designing a skeleton (i.e., class) highlighting the key points of the main body joints. This skeleton is used to correctly annotate positions in the images and enable to the model to learn the keypoints.

The next step is the dataset creation: to build the dataset, we crawled a wide variety of images depicting people in various poses from the web from different sources (to guarantee data heterogeneity). Once the images were collected, we performed a manual annotation task: the “Skeleton” was manually overlaid on each image, carefully placing the key points on the corresponding joints. This time-consuming task ensures that each keypoint is accurately aligned, providing a robust foundation for training the HPE model.

To increase the dataset size, two main approaches are employed: preprocessing and augmentation.

Preprocessing involves performing a series of operations on the dataset images to improve their quality. This includes resizing, pixel normalization, and background removal. On the other hand, augmentation is used to expand the number of available images in the dataset. Techniques such as rotation, translation, and brightness variation are applied to generate augmented images. This helps enhance the performance of the model during training.

After completing all steps, the dataset is created, containing a set of images and for each image a text file with the related labels and keypoints.

Subsequently, the dataset is divided into three datasets:

- the training dataset (train),
- the validation dataset (valid),
- the testing dataset (test).

The training dataset represents the largest portion of the dataset and is used to train the model.

The validation dataset is used to monitor the model’s performance during the training process.

Finally, the testing dataset evaluates the final performance of the trained model.

### 2.2. Training

The second phase of the proposed method for HPE is represented by the training of the HPE model, as shown from the workflow in Figure 2.

In this phase, the model is trained using the training dataset, learning to identify image features and associate them with their respective labels. In this paper, we consider a pre-trained version of the You Only Look Once (YOLO) model (version 8) [13], adapted for HPE tasks.

We resort to the YOLO model primary for its real-time performances in inference; as a matter of fact, by detecting objects in a single pass, it processes images and video streams faster than many other models [14]. This efficiency makes it well-suited for real-time applications where quick response is essential, such as sports analytics and surveillance.

The YOLO model we consider is composed of three main components: parameter settings, a feature-extraction backbone, and a multi-scale prediction head. We configured the model to detect a single object class and regress 25 keypoints, each represented by (x,y,visibility). Compound scaling coefficients are specified for the nano variant (i.e., YOLO8n) of the YOLO model, aimed to balance the model depth, width, and computational efficiency for real-time tasks.

Figure 3 illustrates the architecture of the adopted model, which is composed of a backbone, a neck, and a pose head. The backbone receives the input image and progressively extracts hierarchical visual features through a series of convolutional and C2f blocks, reducing the spatial resolution from stages P1/2 to P5/32 while increasing semantic richness. An SPPF module at the deepest layer captures multi-scale contextual information and expands the effective receptive field.

As shown from Figure 3, the neck aggregates and fuses features across several scales by adopting a combination of upsampling, concatenation, and C2f refinement blocks. These operations integrate high-resolution structural details with low-resolution semantic features, producing three multi-scale feature maps corresponding to P3 (small), P4 (medium), and P5 (large).

Finally, the pose head receives the fused feature maps and generates the final keypoint predictions across all three scales. This design enables accurate human pose estimation by leveraging both fine-grained spatial cues and deep semantic representations extracted throughout the network.

With the aim to provide more details related to the model implementation and for replication purposes, in Listing 1 we show the code snippet related to the YAML (a data serialization format) configuration file related to the adopted YOLO model for pose estimation.

**Listing 1:** YAML Code snippet related to the YOLO model YAML configuration file.

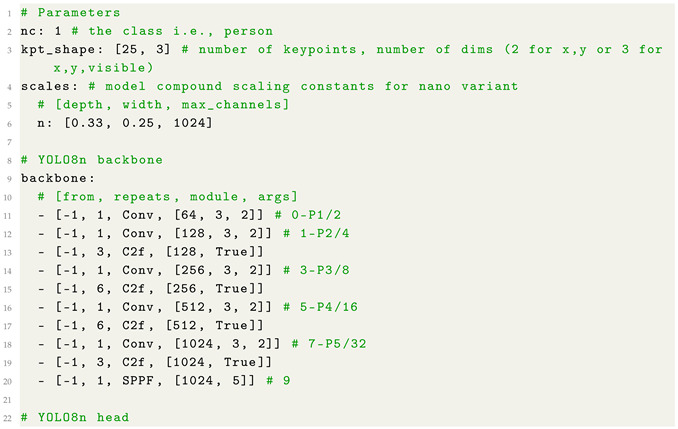



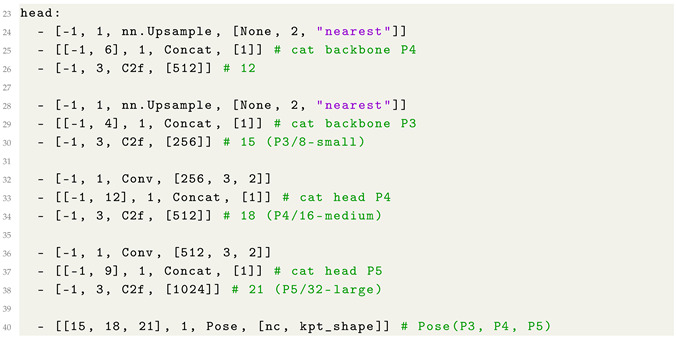



As shown from Listing 1, the backbone consists of a sequence of convolutional layers and C2f modules that progressively downsample the spatial resolution while enriching semantic features, forming a hierarchical pyramid of feature maps from stages P1/2 to P5/32. Convolutional blocks expand the channel dimensions, while the C2f modules, that are based on cross-stage partial connections, are aimed to enhance gradient flow and to reduce computational cost. At the deepest layer, a Spatial Pyramid Pooling-Fast (SPPF) module aggregates multi-scale contextual information to increase the receptive field without introducing significant latency.

The prediction head reconstructs higher-resolution representations through the adoption of successive upsampling and feature-concatenation operations, fusing information from both the backbone and intermediate head outputs to strengthen multi-scale representations. C2f refinement layers at each scale (P3, P4, and P5) further process the fused features, while stride-2 convolutional layers ensure the correct dimensional transitions between pyramid levels. Finally, feature maps from the three scales are jointly passed to the Pose head, which produces the multi-scale keypoint predictions.

Thus, the explained model adapted for HPE task is pre-trained on two datasets i.e., COCO8-Pose and HandKeypoints. The first one includes a class with 17 keypoints for human pose estimation, while the second one is specific to hand pose recognition, using 21 keypoints.

Thus, starting from the pretrained model by utilizing the created skeleton, we perform additional training on the dataset we built in the previous step. This approach leverages the knowledge already acquired by the YOLO model, thereby enhancing the efficiency of human pose detection. The skeleton serves as a reference framework, defining the keypoints and their connections, which helps to accelerate the training process.

Proper optimization of hyperparameters enables the model to learn more effectively from the data. This process ensures that it performs well not only on the training data, but also on unseen data. Once training is complete, the final skeletal model is obtained. This model represents the human pose and adapts to movements.

During this evaluation phase, performance metrics, such as accuracy and loss, are calculated to monitor the model’s ability to correctly recognize human poses and minimize errors. After each epoch, the model’s weights are updated to improve its performance on these metrics. The training cycle continues in this manner for all remaining epochs, with ongoing refinements to the model, until it converges to an optimal configuration.

Subsequently, global metrics are applied to verify the ability of the model to accurately recognize the pose and individual body parts in various positions and conditions.

### 2.3. Testing

The third phase of the proposed method is represented by the testing of the HPE model built in the previous phase, the workflow for which is shown in Figure 4.

Testing is used to evaluate the model’s performance on a testing dataset, which has never been seen by the model during training and validation.

As a matter of fact, after training, the model is tested on the testing dataset, which contains images and videos specifically annotated to assess its performance on new data.

During this process, the model detects keypoints and their coordinates, which are saved alongside the images. The resulting images display the skeletal model overlaid, along with the key points’ coordinates, enabling both visual and quantitative accuracy evaluation.

Moreover, testing can also be performed in real time using a video feed from a camera. This mode allows live monitoring of poses, useful for applications such as motion analysis and exercise assistance.

### 2.4. Pose Estimation and Angle Computation

Once the training and testing of the proposed model are completed, the obtained results can be utilized for several applications. The workflow related to this phase is shown in Figure 5.

The HPE model, starting from an image or a video under analysis, is aimed to predict the keypoint annotations that are saved as .txt files containing the positions of the keypoints in the submitted image/video.

Each annotation file corresponds to an image from the testing dataset and includes the coordinates of the keypoints identified by the model.

These files enable the skeleton building, a skeletal structure that represents the human body. The skeleton building visualizes the entire body configuration and establishes the connections between joints.

Finally, using the skeleton building and the keypoint coordinates, it is possible to compute the angles between joints. These angles are essential for analyzing various movements and postures.

In particular, we consider a Python script developed by the authors, by considering the Python 3.9 version, which aimed to:

- load an input image and a corresponding keypoint .txt file located in a keypoint folder;
- convert normalized coordinates of keypoints to pixel positions on the image;
- plot the keypoints and skeleton, using different colors for body parts (e.g., arms, legs, torso);
- interactive angle computation: when the user clicks near a keypoint, it highlights related joints and displays the anatomical angle (e.g., elbow flexion, shoulder abduction).

With regard to the angle computation, we consider the function provided in Listing 2, aimed to compute the geometric angle (in degrees) formed at a central point p2, based on two line segments: one from p2 to p1 and another from p2 to p3.

**Listing 2:** Python Function for angle computation.

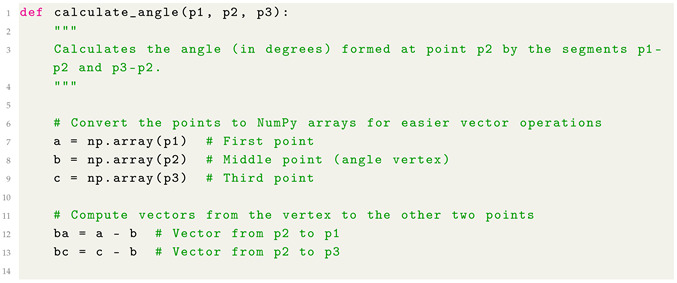



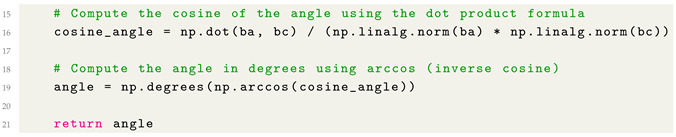



As shown from the function defined in Listing 2 for angle computation, this function computes the geometric angle (in degrees) formed at a central point p2, based on two line segments: one from p2 to p1 and another from p2 to p3.

Below, we describe how it works for joint angle computation from a mathematical point of view:

1. the function takes three 2D points p1, p2, and p3, each represented as (x,y) tuples.
2. it constructs two vectors: $\vec{ba}$ (from p2 to p1) and $\vec{bc}$ (from p2 to p3).
3. using the dot product formula, it calculates the cosine of the angle between the vectors:
  $$\cos\left(\theta \right)=\frac{\vec{ba}\cdot \vec{bc}}{∥\vec{ba}∥∥\vec{bc}∥}$$
4. it then applies the inverse cosine function arccos to get the angle in radians.
5. finally, the result is converted from radians to degrees.

## 3. Experimental Analysis

In this section, we present the experimental analysis aimed to demonstrate the effectiveness of the proposed method for automatic HPE and joint angle computation.

### 3.1. Dataset

As introduced in the previous section, in order to compose an heterogeous dataset, we collected several images depicting people in various poses, with a particular focus on yoga postures and physical exercises. This approach ensured a meaningful variety of human body configurations, which is essential for training a generalizable HPE model.

The dataset was built by combining several images from various publicly available datasets on the Roboflow (https://roboflow.com/, accessed on 30 March 2026) web platform, with additional images gathered from the web from authors.

The Roboflow datasets consulted include following datasets (freely available for research purposes):

- Yoga Pose Computer Vision Project (https://universe.roboflow.com/new-workspace-mujgg/yoga-pose, accessed on 30 March 2026)
- Yoga Pose by Shivam Shrivastav (https://universe.roboflow.com/shivam-shrivastav-jomi6/yoga-pose-apdeo, accessed on 30 March 2026)
- Object Detection Computer Vision Project (https://universe.roboflow.com/mayank-pokhriyal/object_detection-3rcd5, accessed on 30 March 2026)
- YOGA by Vantan (https://universe.roboflow.com/vantan/yoga-ns0by)
- LSP Dataset–Human Detection (https://universe.roboflow.com/naghamxd/lsp-dataset-human-detection, accessed on 30 March 2026)
- Gym or Yoga (https://universe.roboflow.com/1st/gym-or-yoga)
- Group 3 Workout/Exercises (https://universe.roboflow.com/technological-institute-of-the-philippines-i41no/group-3-workout-exercises-tgtls, accessed on 30 March 2026)
- Gestures (https://universe.roboflow.com/umpire/gestures-sdgna, accessed on 30 March 2026)
- Exercise-Pose (https://universe.roboflow.com/highlight-84ih7/exercise-pose, accessed on 30 March 2026)
- Squat (https://universe.roboflow.com/project-pswjo/squat-wv3cn)
- Barbell Detection 2 (https://universe.roboflow.com/jong-o8aun/barbell-detection-2, accessed on 30 March 2026)
- Barbel Tracking (https://universe.roboflow.com/acwsm-naver-com/barbel-tracking, accessed on 30 March 2026)
- Capstone Project (https://universe.roboflow.com/bismillah-4byr6/capstone-project-snjic, accessed on 30 March 2026)
- Exercise (https://universe.roboflow.com/tim-4ijf0/exercise-gciss, accessed on 30 March 2026)
- Workout (https://universe.roboflow.com/danang-university-of-science-and-technology-75xzh/workout-alzp1, accessed on 30 March 2026)

This mixed approach combining several datasets belonging from different sources (and, thus, with images acquired in different conditions) resulted in a varied and representative dataset, tailored to the needs of HPE in both static and dynamic body configurations.

After identifying and inspecting the images, a custom annotation class named Skeleton was developed.

**Figure 6:**
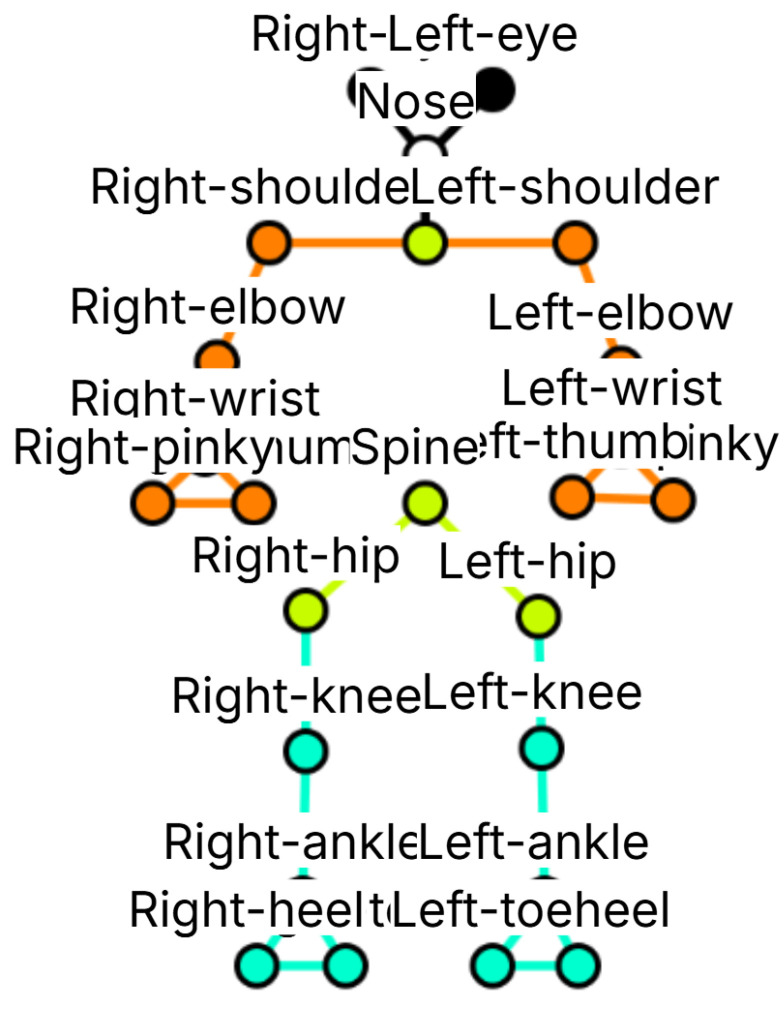
The proposed skeleton with the related keypoints.

As shown from Figure 6, this skeletonis composed of 25 keypoints representing the main joints of the human body, itemized below:

- 0: right eye
- 1: nose
- 2: left eye
- 3: neck
- 4: right shoulder
- 5: right elbow
- 6: right wrist
- 7: right thumb
- 8: right pinky
- 9: left shoulder
- 10: left elbow
- 11: left wrist
- 12: left pinky
- 13: left thumb
- 14: spine
- 15: right hip
- 16: left hip
- 17: right knee
- 18: right ankle
- 19: right toe
- 20: right heel
- 21: left knee
- 22: left ankle
- 23: left heel
- 24: left toe

The skeleton was defined with 25 keypoints, with indices ranging from 0 to 24.

This aspect represents one of the most distinctive features of the proposed method; as a matter of fact, the HPE model typically considers 17 keypoints, and this is reflected in the loss of fundamental information, providing a less accurate analysis.

The proposed 25-keypoints skeleton has been designed to provide a more detailed and anatomically meaningful representation of the human body, particularly suited for the analysis of posture and movement. In particular:

- it provides greater detail in the hands and feet, allowing a more accurate assessment of balance and limb coordination;
- it includes facial keypoints (eyes and nose), enabling the estimation of head orientation and facial alignment, which can be relevant in rehabilitation contexts;
- it distinguishes between the neck and the spine, which may have different angular orientations and therefore should be treated separately for accurate posture evaluation. Unlike the COCO Keypoints dataset [15], which defines 17 keypoints including the neck but not a separate point for the spine or chest, the proposed model enables a finer posture analysis.

Finally, each selected image was manually annotated by overlaying the skeleton, resulting in a labeled dataset consisting of 866 images.

### 3.2. Dataset Augmentation and Preprocessing Techniques

To increase the number of images belonging to the analyzed dataset, various preprocessing and image augmentation techniques were applied, with the aim of generating multiple variations of the same data and subsequently merging them into a single dataset.

**Figure 7:**
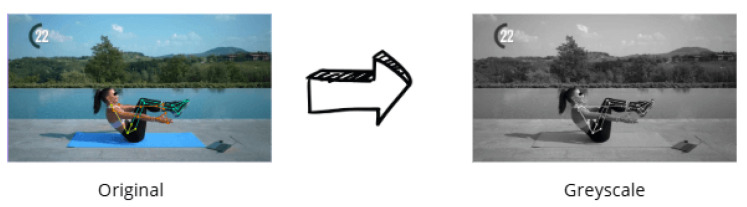
An example of application of greyscale data augmentation.

Different preprocessing strategies were employed, both on the original dataset and on grayscale-converted images, as shown in Figure 7.

**Figure 8:**
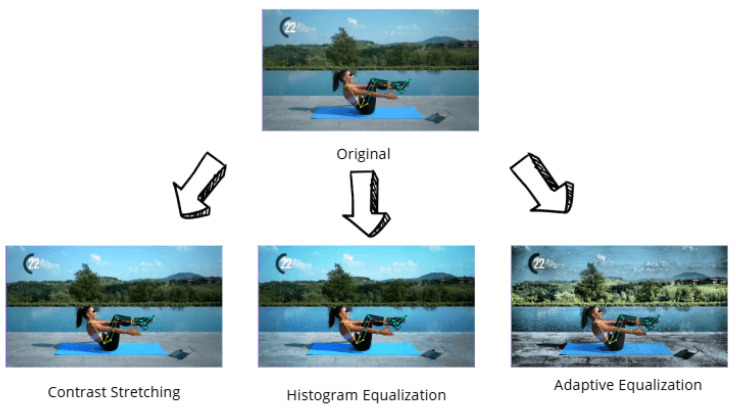
An example of application of auto-adjust contrast data augmentation.

Moreover, auto-adjust contrast techniques were applied, as shown in Figure 8, consisting of three variants:

- Contrast stretching, used to expand intensity values and enhance brightness;
- Histogram equalization, to standardize grayscale levels across the image;
- Adaptive equalization, which focuses on local areas to optimize contrast selectively.

**Figure 9:**
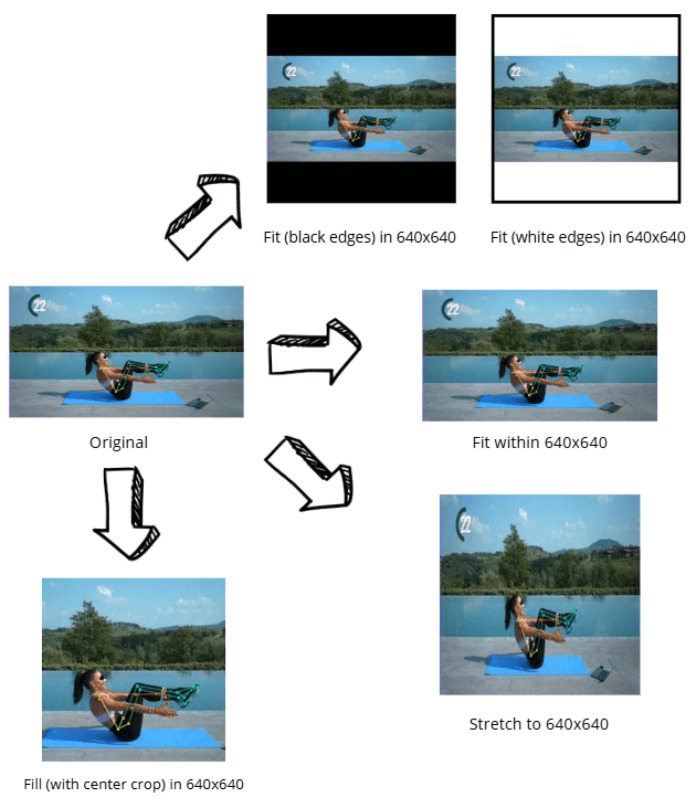
Resize techniques.

Another employed technique is represented by image resizing (Figure 9), in order to standardize the resolution to 560 × 140 pixels. Several strategies were tested, including stretch, fill with center crop, and fit with black or white padding to preserve the original aspect ratio.

**Figure 10:**
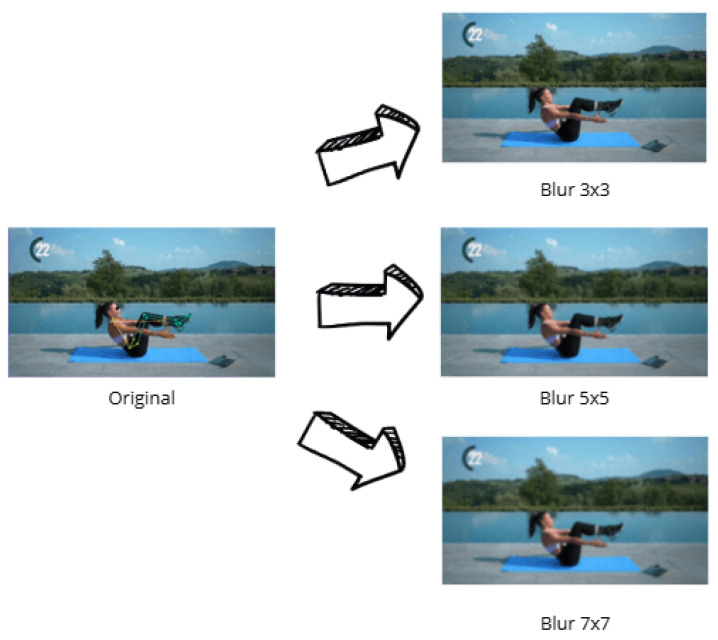
Blur technique.

To simulate varying image quality and enhance the model’s robustness to real-world scenarios, blurring filters (Figure 10) were applied using kernels of sizes 3 × 3, 5 × 5, and 7 × 7.

Additionally, several augmentation techniques were used to further increase the dataset size and improve the model performance. These included:

- Grayscale transformation, applied to 15% of the images randomly during training to reduce reliance on color cues. Unlike grayscale preprocessing, this method is only applied during training and not on the full dataset.
- Hue variation, with a range from −15% to +15%, randomly alters color tones, encouraging the model to focus on shapes and edges rather than color-specific features.
- Saturation adjustment, within a range of −25% to +25%, changes the intensity of colors. This helps the model adapt to conditions like white balance shifts, lighting variations, or weather phenomena (e.g., fog)
- Brightness adjustment, ranging from −15% to +15%, modifies the overall light intensity uniformly across all pixels.
- Exposure variation, from 0.10% to 10%, adjusts the balance between highlights and shadows, simulating more realistic photographic effects.
- Blurring, using a fixed blur of 2.5 pixels, was also introduced to test model performance on less sharp images.
- Noise injection, applied to 0.1% of the pixels, aimed to improve the model’s resilience to random disturbances.

Special attention was also given to the bounding box, the area of interest surrounding the subject in each image. Specific transformations were applied to simulate variations in lighting conditions, including:

- Brightness adjustments, from −15% to +15%, to evaluate the model under both stronger and dimmer lighting;
- Exposure modifications, from −10% to +10%, to reflect real-world scenarios where external lighting influences the visibility of the subject’s region of interest.

Thanks to these techniques, the dataset became significantly more diversified, heterogeneous, and representative. This process not only expanded the dataset but also enhanced the model’s ability to generalize in previously unseen data.

**Figure 11:**
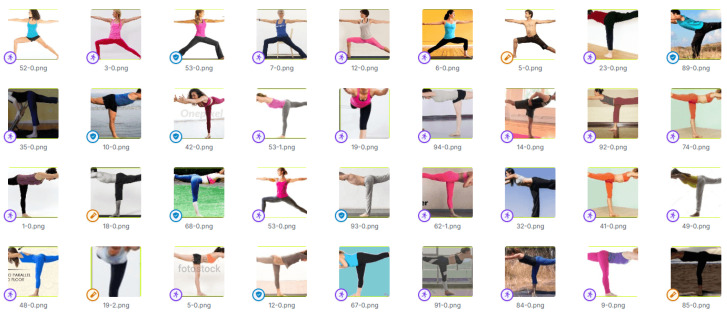
An excerpt of the dataset considered in the experimental analysis.

Thus, the dataset (an excerpt is shown in Figure 11) consists of a total of 152,400 images, divided as follows:

- 133,832 images in the training set, used to train the model;
- 12,300 images in the validation set, used during the training process to monitor learning and prevent overfitting;
- 6308 images in the test set, used for performance evaluation.

In order to replicate the proposed study, we freely release the dataset we built for scientific purposes (https://drive.google.com/file/d/1GNDs1Dafcl0hVWujilrice_0wCBZSclv/view?usp=drive_link, accessed on 30 March 2026).

### 3.3. Experimental Results

To evaluate the performance of the proposed HPE model, we employed a combination of training losses and validation metrics related to object detection and keypoint localization tasks. We compute the set of metrics separately with regard to bounding boxes (*B*) and keypoints (*P*).

The total training loss consists of the following components:

- Bounding Box Loss ($L_{\mathrm{box}}$): Measures the discrepancy between the predicted bounding box $B_{\mathrm{pred}}$ and the ground-truth box $B_{\mathrm{gt}}$ using the Intersection over Union (IoU) metric:
  $$L_{\mathrm{box}}=1-\mathrm{IoU}\left(B_{\mathrm{pred}},B_{\mathrm{gt}}\right),\;\mathrm{where}\;\mathrm{IoU}=\frac{|B_{\mathrm{pred}}\cap B_{\mathrm{gt}}|}{|B_{\mathrm{pred}}\cup B_{\mathrm{gt}}|}$$
- Pose Loss ($L_{\mathrm{pose}}$): Computes the average squared distance between predicted and ground-truth keypoints:
  $$L_{\mathrm{pose}}=\frac{1}{K}\sum_{i=1}^{K}{∥p_{i}-g_{i}∥}^{2}$$
  where $p_{i}=\left(x_{i}^{\mathrm{pred}},y_{i}^{\mathrm{pred}}\right)$ and $g_{i}=\left(x_{i}^{\mathrm{gt}},y_{i}^{\mathrm{gt}}\right)$ denote the predicted and ground-truth coordinates of the *i*-th keypoint, and *K* is the number of keypoints.
- Keypoint Objectness Loss ($L_{\mathrm{kobj}}$): A binary cross-entropy loss indicating the confidence that a keypoint exists at a specific location:
  $$L_{\mathrm{kobj}}=-\left[y\log\left(\hat{y}\right)+\left(1-y\right)\log\left(1-\hat{y}\right)\right]$$
  where y∈{0,1} is the ground-truth presence label and $\hat{y}$ is the predicted confidence score.
- Classification Loss ($L_{\mathrm{cls}}$): A multi-class classification loss based on cross-entropy:
  $$L_{\mathrm{cls}}=-\sum_{c=1}^{C}y_{c}\log\left({\hat{y}}_{c}\right)$$
  where *C* is the number of object classes, $y_{c}$ is the ground-truth label, and ${\hat{y}}_{c}$ is the predicted probability for class *c*.

Each of the losses is computed both on the training set (train/*) and the validation set (val/*).

With regard to evaluation, we consider detection and pose estimation metrics computed on the validation set:

- Precision and Recall:
  $$\mathrm{Precision}=\frac{TP}{TP+FP},\;\mathrm{Recall}=\frac{TP}{TP+FN}$$
  where TP, FP, and FN denote true positives, false positives, and false negatives, respectively.
- Mean Average Precision (mAP):
  - mAP@0.5: Average precision at an IoU (or OKS) threshold of 0.5.
  - mAP@0.5:0.95: Averaged over 10 thresholds from 0.5 to 0.95 with a step size of 0.05:
    $${\mathrm{mAP}}_{\left[0.5:0.95\right]}=\frac{1}{10}\sum_{t=0.5}^{0.95}{\mathrm{AP}}_{t}$$
- Object Keypoint Similarity (OKS): Used to assess keypoint prediction accuracy:
  $$\mathrm{OKS}=\frac{\sum_{i}\exp\left(-\frac{d_{i}^{2}}{2s^{2}k_{i}^{2}}\right)\delta \left(v_{i}>0\right)}{\sum_{i}\delta \left(v_{i}>0\right)}$$
  where:
  - $d_{i}$: Euclidean distance between predicted and ground-truth keypoint *i*
  - *s*: Object scale (e.g., bounding box area)
  - $k_{i}$: Per-keypoint constant controlling the falloff
  - $v_{i}$: Visibility flag of keypoint *i*
  - $\delta (v_{i}>0)$: Indicator function selecting visible keypoints

In the following section, we presents the plots with the explained metrics for each epoch, in particular the metrics/precision(B), metrics/recall(B), metrics/mAP50(B), and metrics/mAP50-95(B) plots refer to the detection metrics computed on bounding boxes using IoU, while the metrics/precision(P), metrics/recall(P), metrics/mAP50(P), and metrics/mAP50-95(P) plots are computed on keypoints using OKS as a similarity measure.

### 3.4. Training and Validation Performance Analysis

**Figure 12:**
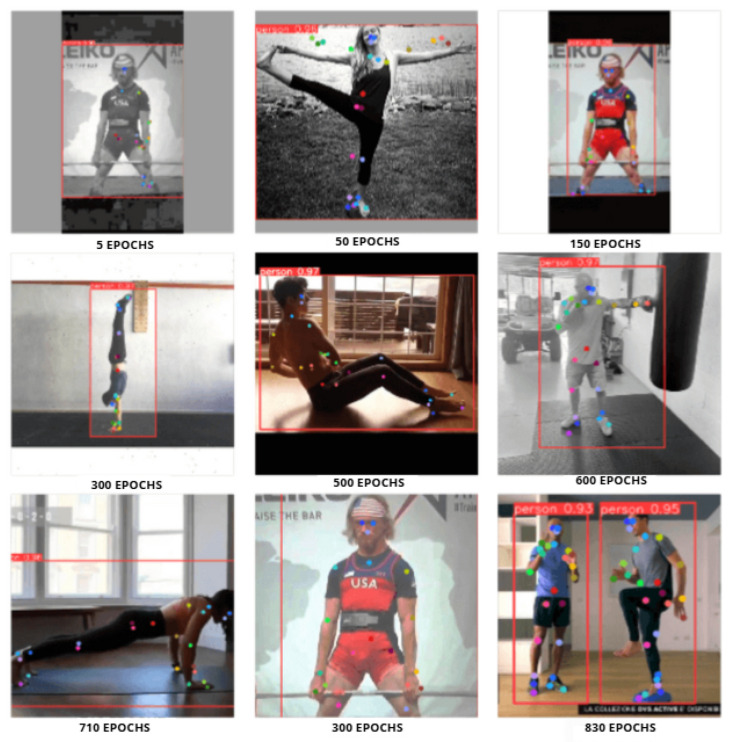
Results over multiple epochs.

The developed HPE model was trained for a total of 830 epochs (Figure 12), and the results from the last 11 epochs were analyzed to evaluate performance.

During the training phase, several plots were generated showing the trends of loss and metric values for both the training and validation phases.

**Figure 13:**
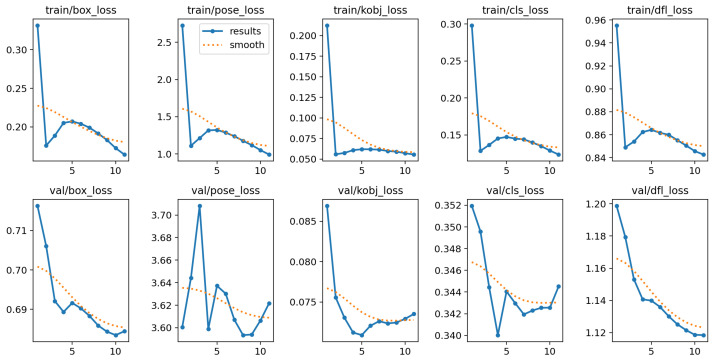
Training and validation loss.

The curves for train/box_loss, train/kobj_loss, and train/cls_loss show a rapid initial decrease, dropping from approximately 0.32, 0.20, and 0.30 in the first epoch to around 0.16, 0.050, and 0.10 by the end of training, respectively (Figure 13). This indicates that the model quickly learns the basic spatial structures.

The train/pose_loss, although starting from a high value (around 2.7), consistently decreases and stabilizes around 1.05. This suggests that pose estimation is inherently more complex, but still effectively addressed.

Similarly, the train/dfl_loss exhibits a steady downward trend from approximately 0.95 to 0.84, maintaining a smooth trajectory without signs of instability.

A gradual, albeit more attenuated, improvement is also observed during the validation phase. The val/box_loss decreases from approximately 0.715 to 0.685, the val/kobj_loss from 0.087 to 0.072, and the val/dfl_loss follows a consistent decline from 1.20 to 1.12. These trends indicate that the model is able to generalize spatial structures to unseen data.

The val/pose_loss and val/cls_loss curves are more noisy. The val/pose_loss fluctuates between 3.60 and 3.72, showing some instability and suggesting that the model struggles more in generalizing pose estimation. This could be due to higher variability or a more complex distribution in the validation dataset. The val/cls_loss also exhibits slight fluctuations between 0.340 and 0.352 but shows an overall downward trend.

**Figure 14:**
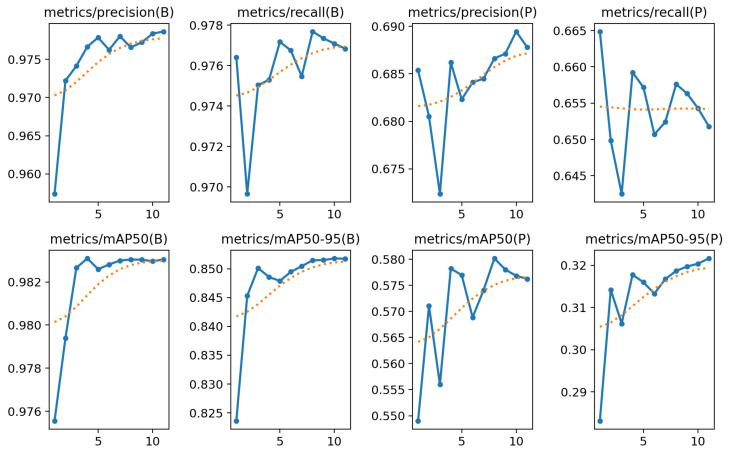
Precision, recall, mAP@50, and mAP@[0.50:0.95].

Figure 14 shows the trends of precision, recall, and mean Average Precision (mAP) metrics, calculated separately for bounding box-based object detection (B) and for pose estimation (P).

The metrics related to object localization via bounding boxes exhibit extremely high and stable performance. Precision(B) and Recall(B) increase rapidly, reaching values of approximately 0.978 and 0.977, respectively, with minimal oscillations and a clear saturation trend. This indicates a well-trained model that is highly reliable in detecting correct objects with few false positives or false negatives.

The mAP@50(B) exceeds 0.983 after just four epochs and remains stable thereafter, while mAP@50:95(B) progressively increases up to approximately 0.851.

In contrast, the metrics related to pose estimation are noticeably lower than those for object localization. Precision(P) and Recall(P) stabilize at approximately 0.688 and 0.653, respectively, but exhibit greater variability. The oscillations in precision suggest a higher instability in discriminating between correct and incorrect poses, while the recall shows a slight downward trend in the final epochs, indicating that some valid predictions may be missed.

The mAP@50(P) shows an upward trend reaching approximately 0.580, but with significant fluctuations. Comparatively, mAP@50:95(P) gradually improves from around 0.28 to approximately 0.32, indicating a slow but steady refinement in the model’s ability to predict accurate poses.

Table 1 and Table 2 summarize the quantitative results for bounding box detection. Over the last 11 epochs, both training and validation losses exhibit a consistent downward trend, indicating progressive convergence of the model. The precision and recall values remain stable around 0.97–0.98, demonstrating strong detection reliability. Similarly, the mAP@50 and mAP@50:95 values, exceeding 0.98 and 0.85, respectively, confirm that the model achieves excellent localization accuracy across multiple IoU thresholds. Overall, the results suggest that the model is effectively learning the spatial distribution of bounding boxes and has reached a point of stable performance with minimal overfitting.

Table 3 and Table 4 report the results obtained for pose estimation. Although the pose loss decreases steadily from 2.72 to 0.99 during training, the corresponding validation loss remains relatively higher, around 3.6, suggesting that the model still faces moderate generalization challenges on unseen data. The precision and recall for pose keypoints stabilize around 0.68, with mAP@50 near 0.58 and mAP@50:95 around 0.32, which are typical values for mid-sized datasets and relatively complex skeleton configurations. These results indicate that while the model captures the main body structure effectively, fine-grained localization of all keypoints could benefit from additional data augmentation or longer training.

### 3.5. Results

The qualitative analysis conducted on the independent test set (without data augmentation) allows for assessing the model’s actual generalization capability across diverse scenarios. In the following images, green circles indicate the ground-truth keypoints, while red crosses represent the model’s predictions.

**Figure 15:**
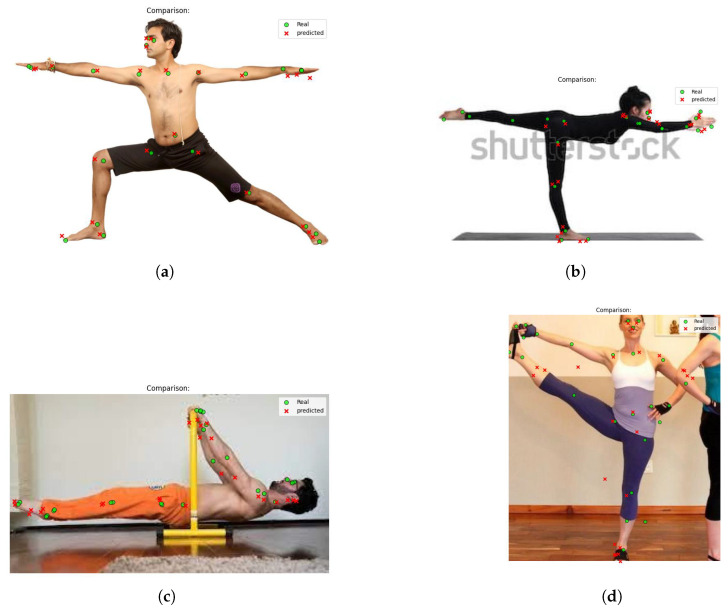
Example of images showing ground-truth and predicted keypoints. (**a**) Ground-truth vs. Predicted Keypoints (Case 1). (**b**) Ground-truth vs. Predicted Keypoints (Case 2). (**c**) Ground-truth vs. Predicted Keypoints (Case 3). (**d**) Ground-truth vs. Predicted Keypoints (Case 4).

Figure 15a, the model shows high accuracy in localizing keypoints. The absence of visual noise and the neutral (white) background facilitate the extraction of anatomical features, confirming the model’s effectiveness under controlled conditions. Although the background remains neutral, Figure 15b highlights a difficulty in correctly predicting the keypoints of the raised leg. This suggests that, for certain specific poses, the model struggles to maintain the geometric consistency of the limb, despite performing well on the rest of the body.

In Figure 15c, the model demonstrates reasonable robustness even in the presence of linear disturbances. The partial occlusion caused by the yellow bar does not prevent the correct identification of most keypoints, indicating a good ability to infer body position “behind” the object. In Figure 15d, the model encounters the greatest difficulties. The combination of a highly complex pose and the presence of “social noise” (another person partially visible in the background) creates spatial ambiguity, leading to less accurate predictions.

These tests show that, although the model is robust in standard scenarios, its reliability remains sensitive to pose complexity and the presence of distracting elements in the visual field.

**Figure 16:**
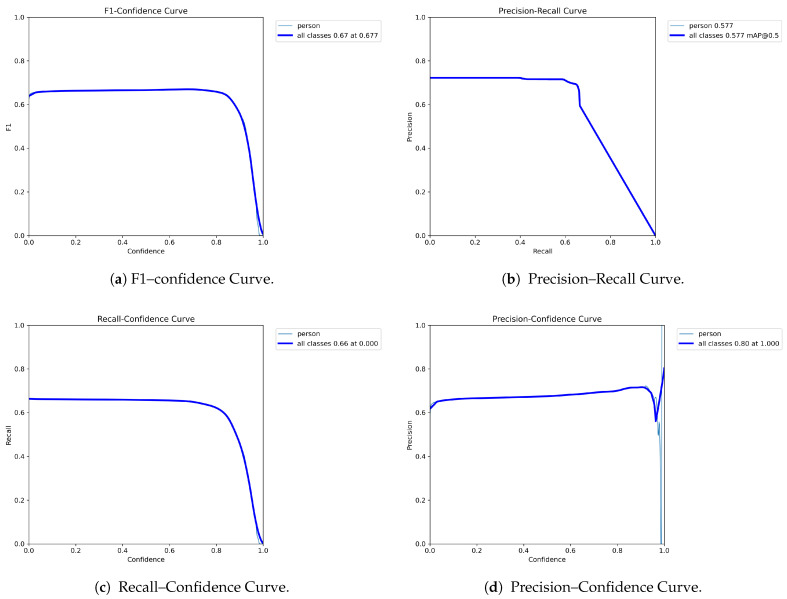
Model’s performance.

The graphs shown Figure 16 in the figure provide an overall assessment of the model’s performance.

Figure 16a reports an F1-score of 0.67, indicating a reasonable balance between precision and recall. This result is satisfactory under standard conditions, but it highlights certain limitations in more complex scenarios.

Figure 16b shows a mAP@0.5 value of 0.577. This metric indicates a moderate ability of the model to correctly localize all anatomical keypoints within an acceptable tolerance threshold.

Figure 16c reports a recall value of 0.66, suggesting that the model fails to correctly identify the complete pose in approximately 34% of the cases. The main issues arise in the presence of occlusions, particularly when the body is partially covered by equipment or by other body parts.

Figure 16d presents the precision–recall curve, which provides insight into the reliability of the model. The presence of a relatively stable plateau between 60% and 80% precision suggests overall robust behavior.

Overall, the obtained results reflect the high complexity of the task, which involves the localization of 25 keypoints. Compared to standard models based on 17 keypoints, this configuration requires greater accuracy in estimating distal extremities (e.g., fingers and feet), which are characterized by small spatial extent and frequent occlusion phenomena. The recall value of 0.66 further suggests a conservative behavior of the model, which tends to avoid predictions under uncertain conditions, particularly in the presence of partial occlusions.

**Figure 17:**
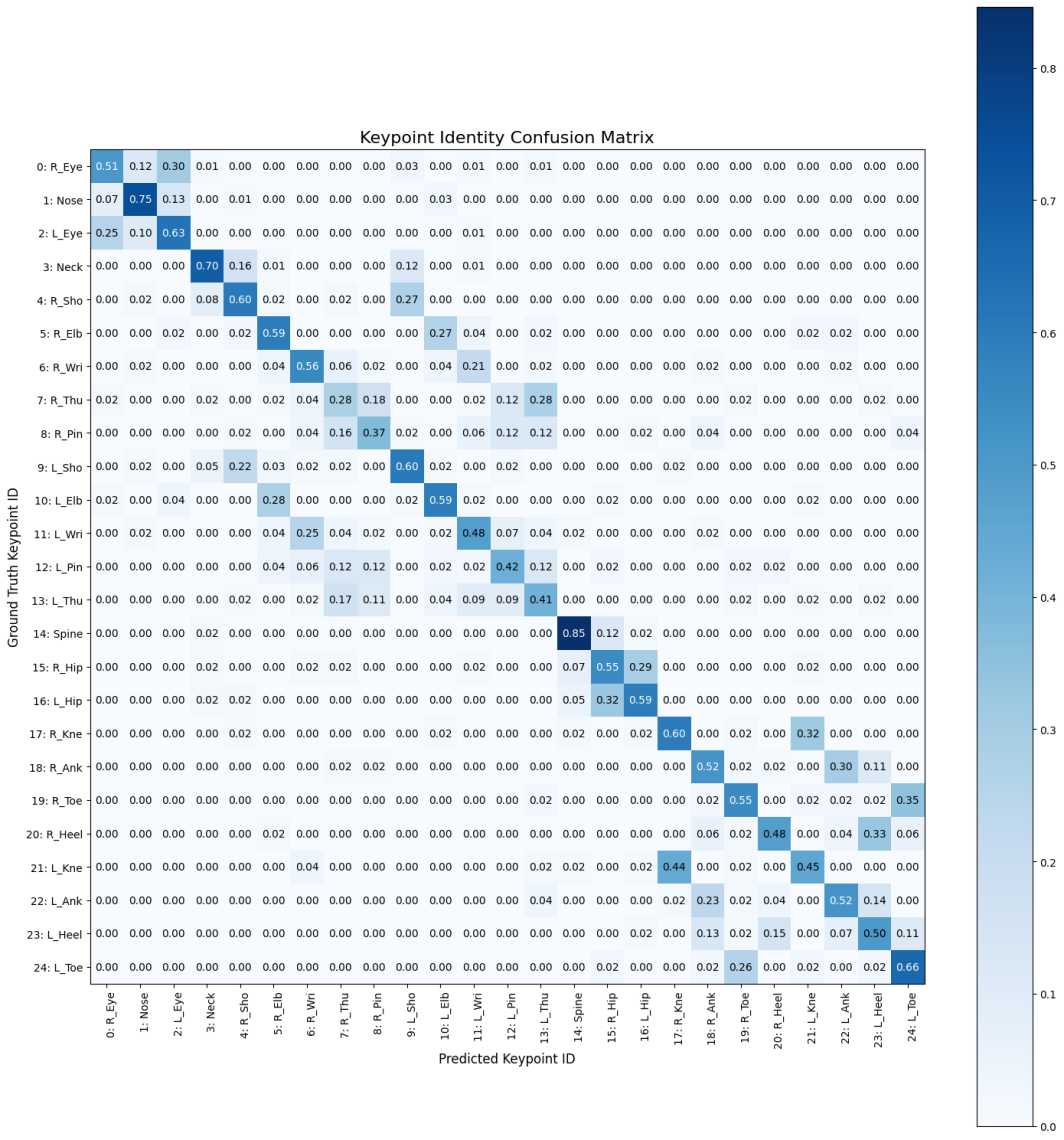
The confusion matrix related to the keypoint-level.

The keypoint-level confusion matrix shown in Figure 17 reveals that central joints, such as the spine and the nose, exhibit high identification stability, whereas distal extremities show significant proximity-related errors. The particular, two main error patterns emerge:

- Symmetry swaps (left–right ambiguity): the visible clusters between R_Hip/L_Hip and R_Eye/L_Eye indicate that the model struggles to correctly handle lateral orientation, especially in inverted or rotated poses.
- Confusion among keypoints within the same anatomical region: the grouping of errors among Thumb, Pinky, and Wrist suggests that the model is able to correctly localize the hand region, but fails to distinguish individual fingers, likely due to insufficient spatial resolution or self-occlusion effects.

These findings help explain the lower mAP value: while the model often correctly detects the subject, it tends to mislabel adjacent or symmetric body parts.

### 3.6. Examples of Application of the Proposed HPE Model

To evaluate the practical applicability of the model, in the following we provide several examples of joint angles computed during a dedicated testing phase using a custom Python script applied to a set of previously unseen images.

**Figure 18:**
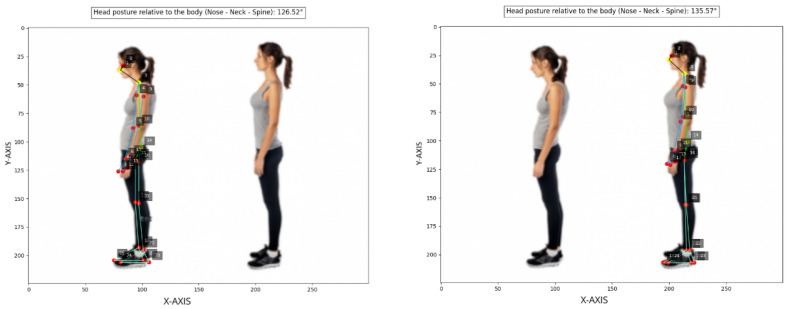
Example of output: angle calculated (test set, no data augmentation).

Figure 18 shows the output of the script, where the angle between three selected keypoints on the image is displayed.

**Figure 19:**
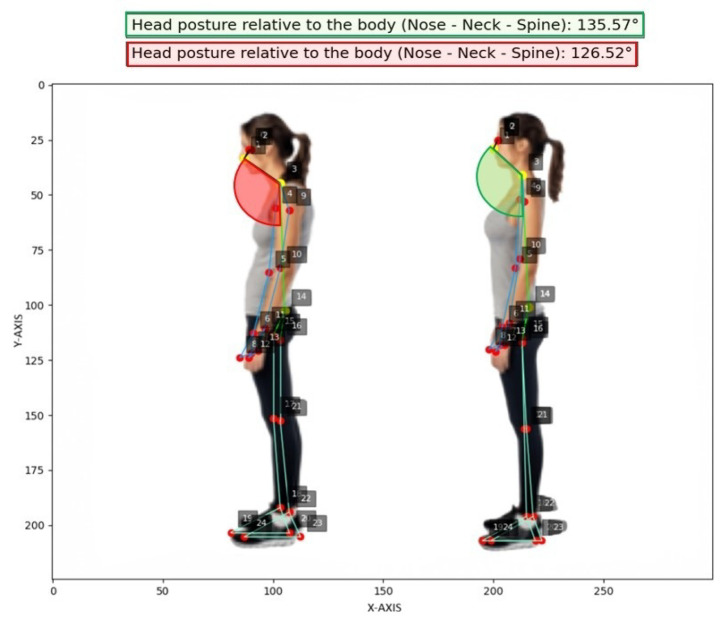
Head posture relative to body angle calculated (test set, no data augmentation).

Differently, (Figure 19) illustrates both an ergonomically correct and an incorrect neck posture relative to the vertical axis, as confirmed by the calculated angular values of approximately 135.57° for the correct posture and 126.52° for the incorrect one (nose, neck, spine).

**Figure 20:**
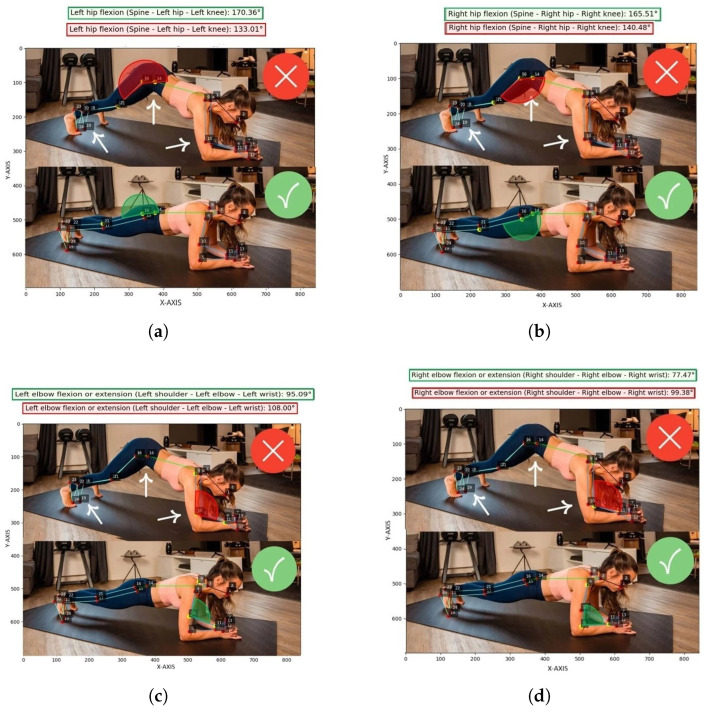

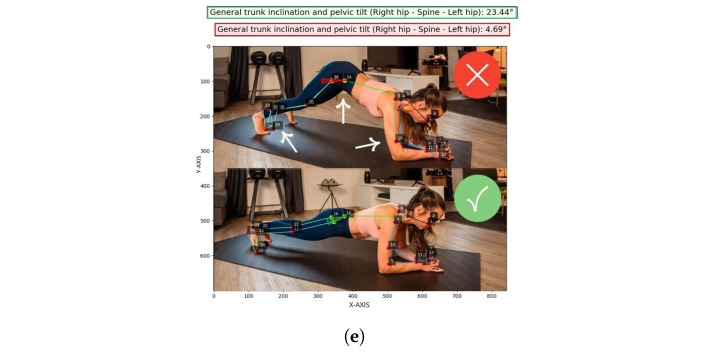
Correct and incorrect plank execution (test set, no data augmentation). (**a**) Left hip flexion angle calculated (test set, no data augmentation). (**b**) Right hip flexion angle calculated (test set, no data augmentation). (**c**) Left elbow flexion angle calculated (test set, no data augmentation). (**d**) Right elbow flexion angle calculated (test set, no data augmentation). (**e**) General trunk inclination angle calculated (test set, no data augmentation).

Additional examples, shown in Figure 20, concern the execution of the plank exercise. Various joint angles were analyzed, including lateral trunk inclination, pelvic tilt, elbow extension (both right and left), and hip flexion. The comparison between correct and incorrect forms reveals significant angular discrepancies, suggesting that the model can effectively identify misalignments and deviations from proper execution.

**Figure 21:**
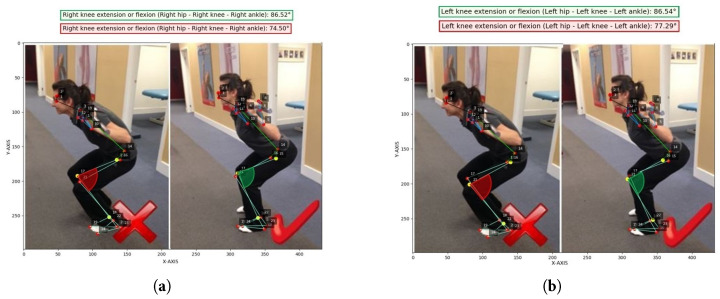
Correct and incorrect squat execution (test set, no data augmentation). (**a**) Right knee flexion angle calculated (test set, no data augmentation). (**b**) Left knee flexion angle calculated (test set, no data augmentation).

Similarly, Figure 21 shows the squat exercise highlight subtler yet biomechanically non-negligible angular differences between the two postural variations.

These discussed examples of inference demonstrate the effectiveness of the proposed HPE model for real-time posture assessment in clinical and athletic contexts, where accurate detection of joint misalignment is essential.

### 3.7. Model Comparison

In this section, with the aim to provide more evidence of the effectiveness of the proposed HPE model, we provide a comparison of the proposed method with respect to the pre-trained version of the YOLO8n model.

**Figure 22:**
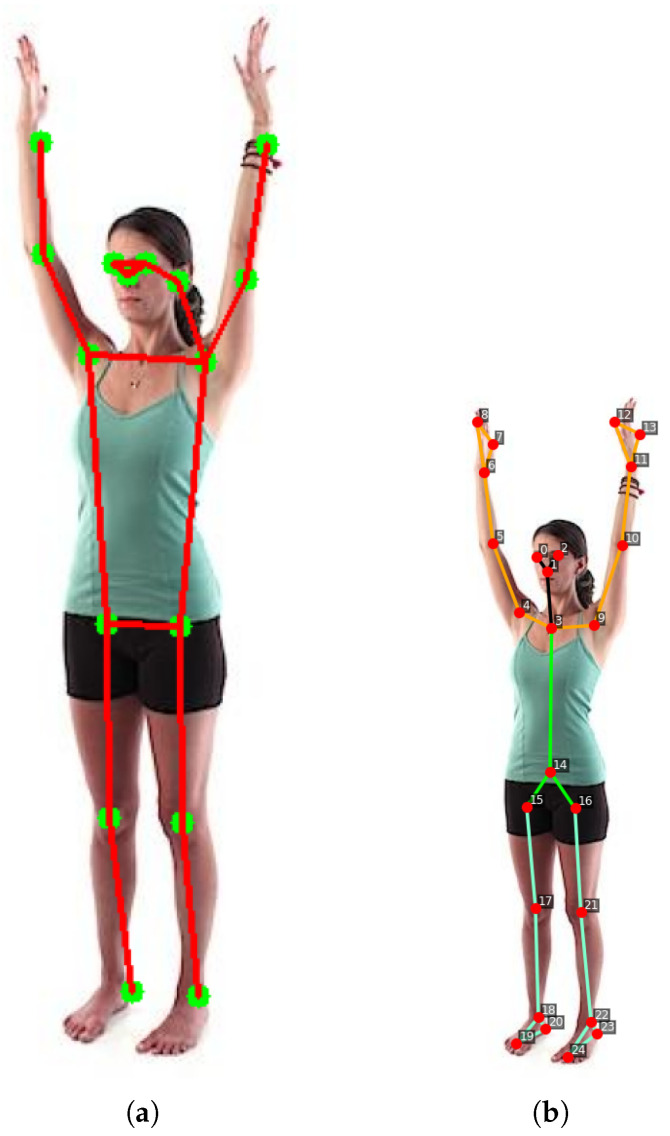
Comparison between models. (**a**) Skeleton output from YOLOv8n. (**b**) Skeleton output from our model.

As a matter of fact, by analyzing the two images shown in Figure 22, it is clear how the number of keypoints directly affects biomechanical precision.

In the skeleton generated by YOLO8n as shown in Figure 22a, the hand and ankle are represented by a single point each. In contrast, our proposed model Figure 22b includes additional keypoints on the palm and fingers, as well as on the tip and heel of the foot. This allows not only the localization of arms and legs but also the interpretation of more complex movements, such as gestures or grips. Such details are crucial in medical rehabilitation and sports applications, for instance, to analyze foot propulsion or wrist rotation. Regarding the core, YOLOv8n Figure 22a defines only a rectangle between the shoulders and hips, while our model Figure 22b establishes a central axis. The presence of points along the midline enables precise calculation of torso flexion and rotation. If the person bends or twists, our model captures these movements with geometric accuracy, whereas the standard model only perceives a shortening between the shoulders.

Finally, the proposed HEP model also includes a neck keypoint, which is essential for accurately analyzing head inclination relative to the torso.

### 3.8. Ablation Test

Considering that in the real-world images or video streams can exhibit occluded points, in this subsection we perform some inference tests to evaluate the effectiveness of the proposed method in these situations: for this purpose, we consider an ablation test.

An ablation test involves selectively removing parts of an image, typically by masking them, to analyze how their absence affects model performance. A significant degradation in performance following the removal of a specific region indicates that the region contained critical information for the inference process. While standard evaluation metrics quantify overall accuracy, ablation studies provide deeper insight into the structural robustness of the model.

**Figure 23:**
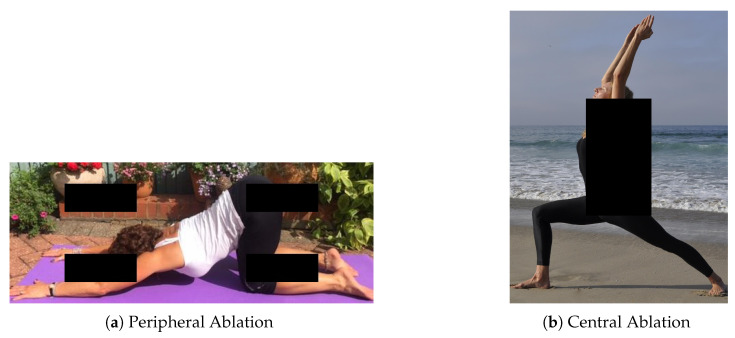
Two ablation scenarios.

To investigate the limits of generalization of the proposed HPE model, two ablation scenarios Figure 23 were designed:

- Peripheral Ablation Figure 23a: Occlusion of the image extremities to evaluate the accuracy of distal keypoints (e.g., hands and feet).
- Central Ablation Figure 23b: Removal of the anatomical core (torso and pelvis) to assess the model’s ability to preserve skeletal consistency using only the visible limbs.

The predictions obtained on the modified images were compared with those generated on the original image. In particular, the Euclidean distance between the predicted keypoints in the two cases was computed using the following formula:

$$d=\sqrt{{\left(x_{\mathrm{true}}-x_{\mathrm{pred}}\right)}^{2}+{\left(y_{\mathrm{true}}-y_{\mathrm{pred}}\right)}^{2}}$$

Finally, the results were visualized using a histogram, showing, for each skeletal keypoint, the percentage error relative to the ground-truth position. The Mean Relative Error (MRE) was also computed.

**Figure 24:**
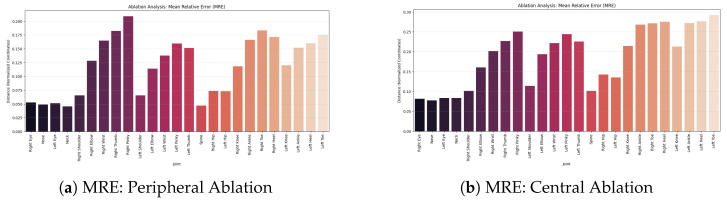
Mean relative error.

The figures present a quantitative analysis of the error (MRE) under two artificial occlusion scenarios. The comparison (Figure 24) between the two plots shows that central occlusion (Figure 24b) is significantly more impactful than peripheral occlusion (Figure 24a), leading to a near tripling of the average error on distal keypoints compared to the baseline. This demonstrates that the model strongly relies on the visibility of the anatomical core to maintain spatial coherence of the limbs.

In detail, as shown in Figure 24a, the proposed HPE model exhibits high robustness in the body’s structural core (Spine MRE: 0.045), while the greatest impact is observed on distal extremities, especially the Right Pinky (0.21) and the Right Toe (0.18). This experiment demonstrates that removing peripheral contextual information selectively degrades the accuracy of keypoints located at the edges of the image, without affecting the stability of the torso.

Conversely, Figure 24b shows a systematic increase in error, with values reaching critical levels in the lower limbs (Left Toe MRE: 0.29 and Left Heel: 0.28). These results confirm that the central region represents a critical information hub for the accurate spatial reconstruction of the entire skeleton.

## 4. Related Work

In this section, the current state-of-the-art related to HPE is discussed. HPE has gained significant traction in the field of computer vision, especially with the advent of deep learning techniques. Traditional methods based on handcrafted features and probabilistic models, such as pictorial structures and graphical models, have gradually been replaced by convolutional neural networks and, more recently, transformer-based architectures [1,3].

A large body of work has explored HPE for clinical and rehabilitation purposes. Sarsfield et al. [4] evaluated depth-sensor-based systems in real-world rehabilitation settings, emphasizing the need for accurate joint tracking for patient recovery. Similarly, Tluli and Al-Maadeed [5] applied machine learning to monitor physiotherapy exercises, showcasing the potential of automated feedback for remote care. In a similar context, recent works such as Alsubai et al. [16] and Gasmi et al. [17] have explored deep learning approaches for human activity recognition and healthcare monitoring, respectively.

Recent surveys have highlighted the role of pose estimation in physical therapy and exercise analysis. Banupriya et al. [6] reviewed deep learning frameworks for rehabilitation-focused HPE, noting the growing use of pose-based metrics for evaluating biomechanical performance. In telemedicine, researchers in. [7] discussed the integration of mixed reality and pose estimation for remote diagnostics and supervision, reflecting the increasing demand for virtual care solutions.

Authors in [18] summarized key 2D deep learning-based pose estimation models, including common architectures, metrics, and datasets. Widely used benchmarks—MS-COCO (17 keypoints), MPII (16), and LSP (14)—mainly target major joints. In contrast, our work employs a 25-keypoint skeleton for more detailed analysis of posture and movement dynamics.

In terms of network architecture, efficient and lightweight models like EL-HRNet have been proposed to enable high-resolution pose estimation on resource-limited devices. These models reduce parameter count while maintaining accuracy, making them suitable for real-time embedded applications [19]. However, they typically focus on joint localization and do not compute angular relationships between keypoints, which are essential for biomechanical analysis.

Moon et al. [20] introduced PoseFix, a refinement network to improve pose estimation accuracy by correcting inaccurate keypoints predicted by base detectors. Similarly, Chen et al. [21] addressed monocular 3D human pose estimation using graph convolutional networks to model skeletal dependencies. However, these works primarily focus on keypoint localization without providing biomechanical analysis such as joint angle estimation.

In addition to spatial pose estimation approaches, recent works such as the Grassmannian-based method for unsupervised pose sequence recognition [22] and variational conditioning of deep recurrent networks for modeling complex motion dynamics [23] have explored spatiotemporal modeling of human motion.

Other methods such as BlazePose [24] and OpenPose [25] offer high-performance pose estimation systems, yet typically rely on a limited number of keypoints (commonly 17 in COCO or 18 in MPII). This restriction constrains their ability to capture subtle biomechanical postural nuances necessary in clinical contexts.

In contrast to these approaches, the proposed method introduces a custom skeleton with 24 anatomical keypoints, designed to capture a more detailed representation of the human body. This allows for improved analysis of joint interactions and posture, especially in physiotherapy exercises. Furthermore, we integrate a geometric angle computation step that quantifies joint angles directly from 2D keypoint coordinates, enabling the detection of postural deviations and incorrect exercise execution in pose estimation pipelines.

Thus, the proposed contribution is aimed to bridge pose estimation with biomechanics through fine-grained keypoint coverage and anatomical angle computation, making it more suitable for physiotherapy, rehabilitation, and remote health monitoring applications.

## 5. Limitations

**Figure 25:**
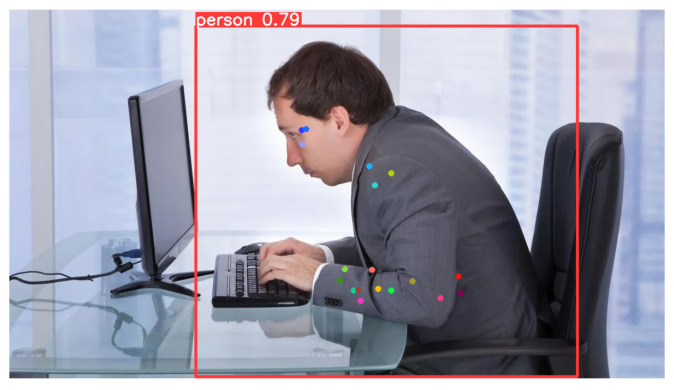
Keypoints related to occlusion and half-body framing affecting pose estimation accuracy.

Despite the promising results, several limitations can be identified in the current approach. First, the model shows reduced accuracy when dealing with images where one leg is raised or partially flexed, as these postures alter the expected spatial configuration of keypoints. As shown in Figure 25, two specific limitations can be observed: the model fails to correctly identify the occluded limb, and the half-body framing of the image further complicates pose estimation by reducing the available contextual information. Finally, the training dataset, although sufficiently large for initial experimentation, does not yet cover the full variability of physiotherapy and sports exercises. Furthermore, the observed mAP@50 value (0.58) should be interpreted in the context of the increased task complexity introduced by the 25-keypoint representation. Compared to standard 17-keypoint setups, the additional keypoints require finer localization, particularly for less visible or occluded joints, which can influence the overall performance metrics.

**Figure 26:**
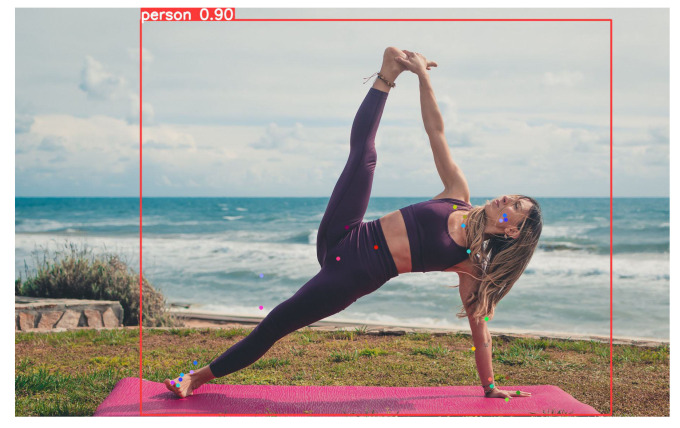
Keypoints related raised-leg posture not correctly recognized by the model.

As also observed in image in Figure 26, the model struggles to correctly recognize poses where the legs are raised. In addition to these limitations, it is also important to consider the evaluation metrics of the model. Another limitation of the proposed approach is the lack of a quantitative evaluation of the estimated joint angles against a reliable ground truth. While the results demonstrate the feasibility of the method through qualitative visualizations, a more rigorous validation using reference measurements (e.g., a goniometer or motion capture systems) would be necessary to assess its clinical accuracy and reliability. This aspect is left for future work. Moreover, since the system estimates joint positions in 2D space, depth-related information is lost, limiting the precision of angular measurements for movements involving significant out-of-plane motion. Another important limitation of the proposed approach is related to the use of 2D joint coordinates derived from a single camera view. This representation is inherently sensitive to projection errors and perspective distortions, which may affect the accuracy of the estimated biomechanical angles. In particular, the same joint configuration can appear different depending on the camera viewpoint, limiting the reliability of angle estimation in out-of-plane movements. Future work may explore the integration of 3D pose estimation techniques, such as lifting networks, or the use of multi-camera systems to mitigate these limitations and improve robustness. Occlusions—such as overlapping limbs or partially hidden body parts—also negatively impact keypoint detection, occasionally leading to incomplete or incorrect skeleton reconstruction.

Expanding the dataset and integrating 3D information would likely improve the model’s generalization and robustness in future developments.

## 6. Conclusions and Future Work

In this paper, we proposed a method for automatic HPE and joint angle computation based on deep learning, with a particular focus on applications in physiotherapy, rehabilitation, and even remote healthcare. By exploiting a custom dataset composed of a wide variety of annotated human poses, along with an extended skeleton incorporating 25 keypoints, we trained a pose estimation model based on the YOLO8n architecture. The proposed method supports not only accurate pose recognition, but also precise geometric angle computation between joints, enabling detailed posture analysis and error detection in exercise execution.

In the experimental analysis, we show the effectiveness of the proposed HPE model by demonstrating that the model performs effectively in both detection and keypoint localization tasks. Specifically, bounding box-based detection metrics show interesting accuracy, while keypoint-based metrics confirm the ability of the model in the recognition of complex joint configurations, albeit with more variability, as shown from the examples of real-world cases. In fact, we showed that the proposed angle computation pipeline provides actionable insight into posture correctness, confirming its suitability for real-time biomechanical assessment.

In future work, we plan to consider different version of the YOLO model with the aim try to increase the obtained performances. Moreover, we will consider the integration in the proposed method of a Large Language Model, aimed to provide textual suggestion related to the correct posture to adopt (in fact, the proposed method is currently able to indicate an incorrect posture, but it does not provide suggestions to correct the posture).

## Figures and Tables

**Table 1:** Training metrics for object detection (bounding boxes) over the last 11 epochs.

Epoch	train/box_loss	train/kobj_loss	train/cls_loss	train/dfl_loss
1	0.33162	0.21249	0.29812	0.95533
2	0.17584	0.05574	0.12851	0.84904
3	0.18878	0.05740	0.13654	0.85406
4	0.20520	0.06075	0.14526	0.86232
5	0.20714	0.06190	0.14732	0.86424
6	0.20406	0.06185	0.14496	0.86166
7	0.19897	0.06135	0.14399	0.85997
8	0.19146	0.05976	0.13972	0.85527
9	0.18299	0.05891	0.13482	0.85057
10	0.17254	0.05705	0.12882	0.84575
11	0.16416	0.05542	0.12348	0.84271

**Table 2:** Validation metrics for object detection (bounding boxes) over the last 11 epochs.

Epoch	Prec(B)	Rec(B)	mAP50(B)	mAP50:95(B)	val/box_loss	val/dfl_loss
1	0.95739	0.97641	0.97556	0.82362	0.71628	1.1987
2	0.97221	0.96966	0.97940	0.84532	0.70609	1.1794
3	0.97413	0.97504	0.98265	0.85013	0.69210	1.1531
4	0.97666	0.97530	0.98309	0.84855	0.68935	1.1407
5	0.97785	0.97718	0.98259	0.84789	0.69163	1.1399
6	0.97625	0.97675	0.98281	0.84947	0.69025	1.1360
7	0.97802	0.97547	0.98299	0.85043	0.68830	1.1302
8	0.97657	0.97769	0.98304	0.85147	0.68587	1.1252
9	0.97723	0.97735	0.98303	0.85150	0.68434	1.1216
10	0.97837	0.97709	0.98296	0.85179	0.68346	1.1187
11	0.97865	0.97684	0.98304	0.85174	0.68443	1.1185

**Table 3:** Training metrics for pose estimation over the last 11 epochs.

Epoch	train/pose_loss	Prec(P)	Rec(P)	mAP50(P)
1	2.7273	0.68537	0.66487	0.54902
2	1.1105	0.68051	0.64983	0.57107
3	1.2131	0.67236	0.64249	0.55598
4	1.3164	0.68620	0.65923	0.57820
5	1.3223	0.68231	0.65718	0.57696
6	1.2865	0.68410	0.65068	0.56885
7	1.2384	0.68449	0.65239	0.57404
8	1.1761	0.68662	0.65761	0.58015
9	1.1187	0.68712	0.65632	0.57802
10	1.0518	0.68946	0.65430	0.57681
11	0.9916	0.68781	0.65179	0.57623

**Table 4:** Validation metrics for pose estimation over the last 11 epochs.

Epoch	val/pose_loss	val/kobj_loss	val/cls_loss	val/dfl_loss
1	3.6006	0.08691	0.35194	1.1987
2	3.6442	0.07555	0.34957	1.1794
3	3.7081	0.07305	0.34444	1.1531
4	3.5990	0.07121	0.34003	1.1407
5	3.6372	0.07089	0.34405	1.1399
6	3.6303	0.07205	0.34297	1.1360
7	3.6071	0.07261	0.34194	1.1302
8	3.5935	0.07234	0.34230	1.1252
9	3.5940	0.07245	0.34255	1.1216
10	3.6064	0.07291	0.34256	1.1187
11	3.6217	0.07351	0.34452	1.1185

## Data Availability

The data presented in this study are available on request from the corresponding author.
